# Supplementary material for: Complexome profiling of the Chlamydomonas psb28 mutant reveals TEF5 as an early PSII assembly factor
Source: Plant Cell. 2025 Mar 17;37(6):koaf055. doi: 10.1093/plcell/koaf055 (PMC12164590; doi:10.1093/plcell/koaf055)
Supplement: koaf055_Supplementary_Data [file koaf055_supplementary_data.zip › psb28_interactive.zip!/plots/intensity/qualityPlots/peptides/FNR1_Cre11.g476750.t1.2.html]

### Cre11.g476750.t1.2

Trivial name: FNR1  
SeqCoverage [median]: 31.20  
Mass: 38.27 kDa  
  
 

| Sequence | MissedCleavage | Mass [Da] | wtIntensity | muIntensity | Score |
| --- | --- | --- | --- | --- | --- |
| ASTAVTTDMSK | 0 | 1110.52 | 2244.56 | 1698.58 | 137.46 |
| ASTAVTTDMSKR | 1 | 1266.62 | 16457.74 | 10766.47 | 142.89 |
| AVYVDPETGK | 0 | 1077.53 | 0.00 | 287.46 | 62.00 |
| AYPGQFR | 0 | 837.41 | 5341.42 | 3163.50 | 98.03 |
| AYPGQFRLDYALSR | 1 | 1655.84 | 0.00 | 2144.98 | 41.64 |
| GMMPGIQDMLER | 0 | 1376.63 | 743.95 | 1865.34 | 83.87 |
| LDYALSR | 0 | 836.44 | 17082.39 | 10936.44 | 120.62 |
| LDYALSREQNNR | 1 | 1477.73 | 421.96 | 0.00 | 43.63 |
| LYDEELQAIAK | 0 | 1291.67 | 17132.26 | 40364.17 | 207.83 |
| LYSIASSR | 0 | 895.48 | 18901.03 | 17628.06 | 116.90 |
| NQWHVEVY | 0 | 1073.49 | 764.62 | 0.00 | 71.72 |
| SVEKITGPK | 1 | 957.55 | 0.00 | 402.84 | 85.73 |
| TVPTKLEEGEMPLNTYSNK | 1 | 2150.06 | 0.00 | 9513.10 | 92.99 |
|  |  |  | 79089.9 | 98770.9 |  |
